# Expanding automated gene summaries for *Caenorhabditis* and parasitic nematode species in WormBase

**DOI:** 10.17912/micropub.biology.001267

**Published:** 2024-07-16

**Authors:** Ranjana Kishore, Valerio Arnaboldi, Wen J. Chen, Paul W. Sternberg

**Affiliations:** 1 Division of Biology and Biological Engineering, California Institute of Technology, Pasadena, California, United States

## Abstract

WormBase and the Alliance of Genome Resources provide several types of gene data including annotations to ontology terms and controlled vocabularies. These are used to automatically generate text summaries to give users a cogent view of gene function. However, automated summaries are not available for genes that lack curated annotations. To increase the genome coverage of the summaries in WormBase, we developed a new software module that generates additional gene summaries for *C. elegans* and new gene summaries for nine other nematode species: four *Caenorhabditis* species ( *C. brenneri, C. briggsae, C. japonica, C. remanei* ), *P. pacificus* , and four parasitic species ( *B. malayi, O. volvulus, S. ratti and T. muris* ).

**Figure 1. Workflow diagram representing the gene summary generation process.**
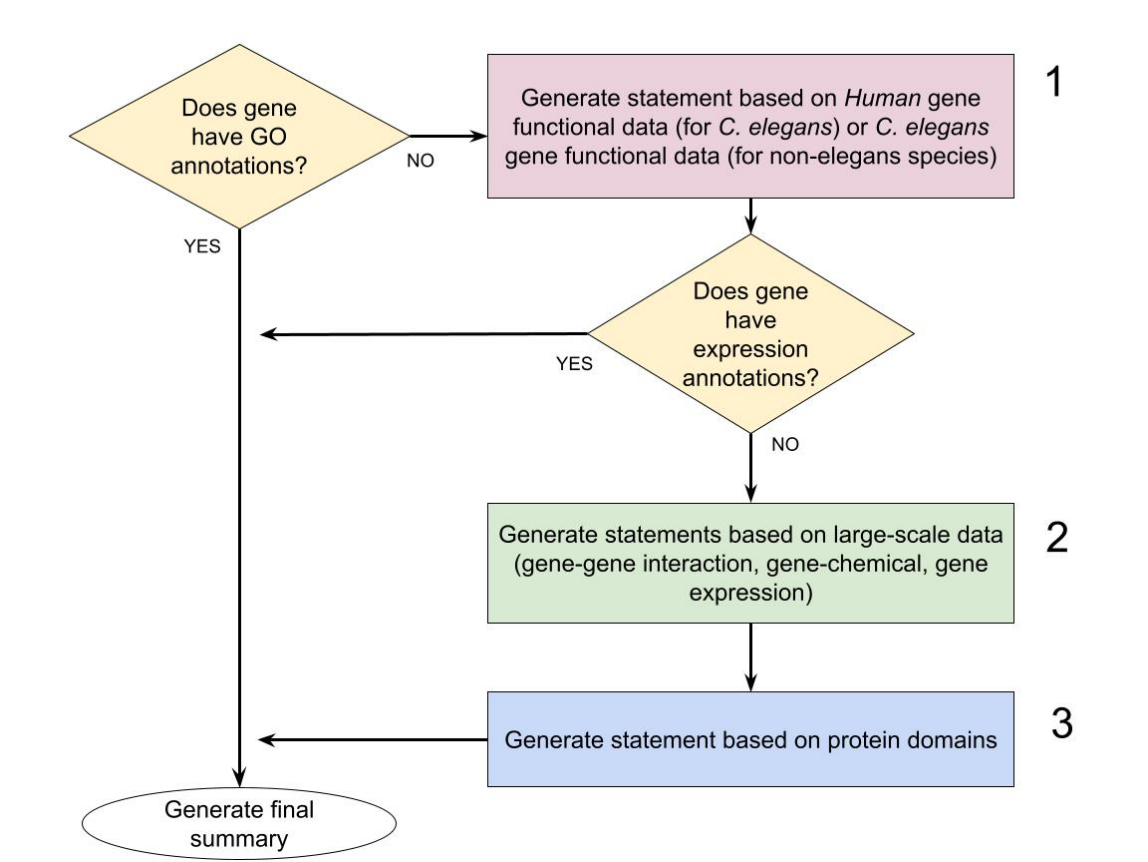
The three strategies used to generate summaries for genes that lack curated functional annotations are shown in steps 1, 2, and 3.

## Description

Short textual gene summaries that describe gene function are valued for the ease with which they convey information about a gene and its biological role. The main advantage of gene summaries is that they require no specialized knowledge of database vocabularies and annotations. For several years, WormBase (Sternberg et al., 2024) has provided manually written gene summaries, and later developed an algorithm in collaboration with the Alliance of Genome Resources (Alliance of Genome Resources Consortium, 2024) to generate automated summaries (Kishore, et. al., 2020) . These automated summaries are based on structured, curated gene annotations to ontologies including the Gene Ontology (GO; The Gene Ontology Consortium, 2023), Disease Ontology (DO; Baron et al., 2024) and gene expression annotations to the WormBase Anatomy Ontology (AO; Lee and Sternberg, 2003). We have recently developed a new WormBase-specific software module based on the algorithm developed at the Alliance to provide additional summaries for genes from *C. elegans* and other nematodes that lack curated functional annotations. This module uses large-scale data from high throughput experiments to generate summaries related to gene expression, gene-gene and gene-chemical interactions. Further, the module uses orthology to transfer gene function statements from related species to the gene of interest in order to build a summary. These strategies resulted in several thousand additional summaries for *C. elegans* genes and new gene summaries for nine other WormBase nematode species (Howe et al., 2012; Howe et al., 2016) . See Table 1 for the full list of species, for numbers related to the different data type statements and the total number of generated gene summaries.

The software module implements the following strategies (depicted in Figure 1 ) in order to generate a gene summary:

**1. Data transfer from orthologous genes.**

(i) For each *C. elegans* gene, human orthologs with the most number of prediction methods reported by WormBase were selected and the associated molecular activity and disease implication was included in the *C. elegans* gene summary. These statements are transferred to the gene summary only when GO data are not present.

Example *C. elegans* act-3 gene summary:

*Expressed in gonad and head. Human ortholog(s) of this gene implicated in several diseases, including Baraitser-Winter syndrome 1; Baraitser-Winter syndrome 2; and autosomal dominant nonsyndromic deafness 20. Human ACTB Contributes to nucleosomal DNA binding activity. Human ACTB enables several functions, including Tat protein binding activity; enzyme binding activity; and kinesin binding activity. A structural constituent of postsynaptic actin cytoskeleton. Is predicted to encode a protein with the following domains: Phosphorylation site; Actin; Actin family; and ATPase, nucleotide binding domain. Is an ortholog of human ACTB (actin beta).*

(ii) For nematodes other than *C. elegans,* the best orthologs were selected from related nematode species based on the number of prediction methods and the number of GO annotations in WormBase, and the associated biological processes were included in the summaries.

Example *C.* *briggsae fem-2* gene summary:

*Predicted to enable protein serine/threonine phosphatase activity. Is an ortholog of C. elegans fem-2. In C. elegans, fem-2 is involved in male sex determination; masculinization of hermaphroditic germ-line; and nematode male tail tip morphogenesis.*

**2. Large-scale data.** Large-scale data such as microarray, tiling array and RNA-seq studies that have been collated and summarized in WormBase (Grove et al., 2018) were used to generate statements related to gene expression and its regulation by chemicals and other genes. These statements are included in the gene summary only when GO and expression data are not present. Example gene summary for *C. elegans* abt-3:

*Enriched in male based on RNA-seq studies. Is affected by several genes including eat-2; sir-2.1; and npr-1 based on RNA-seq; tiling array; and microarray studies. Is affected by seven chemicals including Tunicamycin; manganese chloride; and multi-walled carbon nanotube based on microarray and RNA-seq studies.*

**3. Protein domain data.** Protein domain data from InterPro (Paysan-Lafosse et al., 2022) in WormBase were used to build additional statements for gene summaries. These statements are included in the gene summary only when GO and expression data are not present. Example gene summary for *C. japonica,* Cjp-gid-1:

*Is predicted to encode a protein with the following domains: SPRY domain; B30.2/SPRY domain superfamily; and Concanavalin A-like lectin/glucanase domain superfamily. Is an ortholog of C. elegans gid-1.*

**Table:** 

Nematode species	Genes with summaries (without new module)	Genes with summaries (with new module)	Molecular activity statements (transferred data from related species)	Gene expression statements (from large scale data)	Gene regulation of gene statements (from large scale data)	Chemical regulation of gene statements (from large scale data)	Protein domain statements
							
*C. elegans*	15,075	28,972	103	8,144	12,347	7,513	2,522
*C. brenneri*	22,029	24,278	4,263	NA	NA	NA	9,250
*C. briggsae*	17,117	18,280	3,205	NA	810	NA	6,263
*C. japonica*	17,712	20,214	3,554	NA	NA	NA	7,223
*C. remanei*	22,662	25,896	3,799	NA	NA	NA	10,752
*B. malayi*	9,333	9,674	2,057	392	NA	507	2,775
*O. volvulus*	9,219	9,475	2,051	NA	NA	NA	2,821
*P. pacificus*	12,342	15,294	2,770	58	NA	NA	5,805
*S. ratti*	8,644	9,713	2,040	NA	NA	NA	3,166
*T. muris*	8,300	10,110	2,008	NA	NA	NA	3,662

**Table 1. Types of data-specific statements and numbers of gene summaries in WormBase release version WS292.** NA indicates that the data is not available in WormBase.

## Methods

**Software:** The gene summaries generation software is open source and available at https://github.com/alliance-genome/agr_genedescriptions . The new WormBase module can be found under the “wormbase” folder.

**Viewing gene summaries and availability of data files:** Individual gene summaries can be viewed in the “Overview” widget at the top of the gene pages in WormBase. In addition, data files (txt, json, and tsv formats) are available for download from the curation server at Caltech: https://caltech-curation.textpressolab.com/files/pub/gene_descriptions/ and are organized by WormBase release version. The same files can also be obtained from the official WormBase downloads server at https://downloads.wormbase.org/releases/ , for example: https://downloads.wormbase.org/releases/WS292/species/c_elegans/PRJNA13758/annotation/c_elegans.PRJNA13758.WS292.functional_descriptions.txt.gz
